# Low perceived warmth of AI agents reduces trust towards them

**DOI:** 10.1038/s41598-026-42252-1

**Published:** 2026-03-02

**Authors:** Katarzyna Samson, Tomasz Zaleskiewicz

**Affiliations:** Center for Research in Economic Behavior, Faculty of Psychology in Wroclaw, SWPS University, Ostrowskiego 30b, 53-238 Wroclaw, Poland

**Keywords:** Warmth, Competence, Social cognition, Trust, Trust game, Artificial Intelligence, AI, Human behaviour, Computer science

## Abstract

Artificial intelligence (AI) agents represent a new class of social actors within social and economic systems. To ensure the smooth functioning of human-AI societies, it is crucial to understand how trust between humans and AI agents is developed. The present study (*N* = 400), conducted on a representative sample of U.S. residents, investigated how the fundamental dimensions of social perception may affect differences in trust towards humans and AI agents. We manipulated human and AI trustees’ warmth and competence and measured trust towards them in a trust game. Overall, AI trustees were trusted less than human trustees were, especially in the low warmth conditions. We discuss warmth as a crucial determinant of trust in the context of human-AI interactions and suggest potential implications of these results for designing trustworthy AI systems.

## Introduction

As artificial intelligence (AI) advances, a human-AI society has become a reality. AI technologies continue to evolve and integrate into our daily lives, taking on various forms and fulfilling diverse functions. From being mere tools, these technologies have morphed into autonomous, independent entities operating across a wide range of settings and people perceive and interact with them as real social actors^1–3^.

People commonly experience both apprehension and excitement in regard to different AI technologies^4–7^. They recognize the benefits that AI can offer to individuals, organizations, and society, such as increased efficiency, innovation, effectiveness, better resource utilization, and reduced costs. Many are enthusiastic about AI’s potential to simplify and enhance their daily lives^5–7^. However, people are also aware of the significant risks and challenges associated with AI. These include misuse and abuse, the codification and reinforcement of unfair biases, infringements on human rights such as privacy, the spread of fake content online, deskilling and technological unemployment, and dangers from mass surveillance technologies, critical AI failures, and autonomous weapons^5–8^. The development of AI also poses threats to human autonomy and agency, particularly when decision-making is delegated to code-driven, black-box tools, which can undermine independence, control, and choice^9,10^. The ultimate fear is that machine intelligence could match or surpass human intelligence, potentially leading to scenarios where machines turn against humans, replace them as the dominant “life” form on Earth, or even result in the annihilation of humankind^5,6,11–13^.

These concerns highlight the importance of understanding the dynamics of people’s trust in AI in the context of human-AI interactions, which has recently become a prominent topic of scientific inquiry scattered across numerous fields, with varying methodological approaches and different representations of AI^14–18^.

In the literature on interpersonal trust, trust is generally conceptualized as an attitude of one person (the trustor) toward another (the trustee), who possesses a combination of objective and subjective attributes (as perceived by the trustor) that contribute to their trustworthiness^19^. Interpersonal trust is typically understood as comprising both cognitive and affective components. Cognitive trust reflects the trustor’s confidence in the trustee’s competence, leading to attributions of capability and reliability. In contrast, affective trust is based on the trustor’s belief in the trustee’s benevolent intentions^20^.

Extending the concept of interpersonal trust to AI systems is based on the assumption that AI can functionally assume the role of a trustee, similar to a human counterpart^19^. A key aspect emphasized in the literature on interpersonal trust is the role of vulnerability in trusting relationships. Mayer and colleagues^21^ [p. 712] provide one of the most widely adopted definitions of trust, which we also follow in this work: trust is “the willingness of a party to be vulnerable to the actions of another party based on the expectation that the other will perform a particular action important to the trustor, irrespective of the ability to monitor or control that party.” The notion of vulnerability is particularly relevant in the context of human-AI relationships, as AI systems increasingly influence individuals’ well-being, thereby heightening their exposure to potential risks^19^. This growing dependence on AI further supports the validity of treating AI systems as analogous to human trustees in the study of trust.

Trust in AI in the context of human-AI interactions can be analyzed as operating within a dyadic framework against an interactive context^15,17,18,22^, just like trust in the context of interpersonal interactions^21^ and trust in automation^23^. Stanton and Jensen^22^ proposed to represent trust in AI systems as *T*(*u*, *s*, *a*), where *T* is trust in a system, *u* is the user, *s* is the system, and *a* is the context of the interaction. A meta-analysis of research on trust in AI validated this approach. The antecedents of trust in AI can be classified into three categories: factors related to the human trustor, factors related to the AI trustee, and factors related to the shared context of their interaction^15,17^. Factors related to the human trustor can further be classified into ability-based, of which the most important are competency, understanding, and expertise, and characteristic-based, such as culture and personality. Factors related to the AI trustee are either performance-based, of which the most important are performance and reliability, or attribute-based, such as anthropomorphism, AI personality and behavior, as well as reputation and transparency. Factors related to the interaction include team-related, such as communication, and task-related, such as risk. All these higher-level factors were proven to be significant predictors of trust in AI, although some were shown to have contradictory effects on trust, and interactions between them were rarely addressed in research. Overall, factors related to the AI trustee were shown to have the greatest effect on the human-AI trust^15,17^.

Research on social cognition^24–27^ might be particularly useful for understanding how factors related to the AI trustee affect human-AI trust. In this approach, upon encountering others people must determine, first, their intentions towards them – whether they are a friend or foe – and, second, their ability to act on those intentions. These two are referred to as warmth and competence, respectively, and constitute the two fundamental dimensions of social cognition. Judgments of warmth are driven by the covariation of interests between the perceiver and the perceived or, in other words, the degree to which interaction partners’ intents or goals correspond^3^. In contrast, judgments of competence are determined by attributions of status and autonomy^3^. The combination of low/high warmth and low/high competence is related to emotional and behavioral reactions towards the interaction partner. Evidence suggests that in the case of human trustees, perceptions of trustworthiness and trust depend rather on the trustee’s warmth than on their competence^27^.

AI agents prompt social cognitive judgments similar to those elicited by humans. Previous research has identified warmth and competence as significant dimensions in the perception of AI agents, similarly to the perception of humans^3,28–30^. However, the role of trustee warmth and competence for trust in human-AI interactions is still unclear. A cross-sectional survey identified trustee warmth and competence as two critical determinants of trust in AI^31^. Both competence and warmth have been shown to positively affect perceived trustworthiness and trust in human-robot interactions^32^. A meta-analysis of human-robot interaction studies found that competence-related characteristics have the greatest impact on trust^15^. However, one study also showed that the perceived warmth of the trustee had a stronger effect on perceived trustworthiness and trusting behavior than competence^33^. Additionally, when participants had to choose between a high-competence system and a high-warmth, the majority chose the high-warmth system^34^. These conflicting results suggest that in the case of human-AI relations, the relative importance of warmth and competence for affective and behavioral reactions, such as trust, may differ from their importance in human-human relations.

To date, research directly comparing trust in human-AI interactions with trust in human-human interactions is still scarce and often context-specific, and its results are also conflicting. A study conducted by Zhang et al.^35^ showed that in the context of solving a chess puzzle as a team, people trusted AI teammates more than human teammates and high-competence teammates more than low-competence teammates, be they humans or AI agents. Georganta and Ulfert^36^ showed that when introducing new teammates, perceptions of trustworthiness and affective interpersonal trust were lower in the case of AI teammates than in the case of human teammates, but the levels of cognitive interpersonal trust and trusting behaviors were not different for the two types of teammates. Fahnenstich and colleagues^37^ compared the levels of trust towards human and AI support agents and found that under higher risk, participants exhibited increased trust towards AI trustees, as compared to human trustees, while under lower risk levels, there were no differences in trust levels towards the two types of support agents. Langer and colleagues^38^ showed that when making personnel hiring decisions, initial trust levels, but not perceived trustworthiness or behavioral trust towards a human preselection support agent were higher than towards an automated preselection support agent. Moreover, the violation of trust reduced more strongly the trustworthiness and trust in the case of the human trustee than in the case of the AI trustee.

In the present research, we compared how trustee warmth and trustee competence affected behavioral trust in human-human and human-AI interactions using the trust game methodology^39^, which is widely applied for interpersonal trust research^40^. We hypothesized that (1) in the case of human trust game partners, the trustees’ warmth would more strongly affect trust towards them, while (2) in the case of AI agent trust game partners, trust towards them would be more strongly affected by the trustees’ competence.

## Methods

This research was carried out in accordance with the recommendations of the Declaration of Helsinki with written informed consent from all participants. The protocol was approved by the SWPS University Ethics Committee, decision number 04/P/03/2024.

We measured trusting behaviors in a trust game with real pay-offs using a between-participant design with three factors: trustee type (type of trustee: AI agent vs. human), trustee warmth (warmth: low vs. high), and trustee competence (competence: low vs. high).

### Participants

The study was conducted on an online sample of US residents (*N* = 400; 48.8% male, 51.2% female, age 18–83, M = 45.42, SD = 15.67) recruited on the Prolific platform. The sample was representative with respect to age, sex, ethnicity and political affiliation. The compensation for participating in the study comprised of two elements: base compensation of £0.45 for all participants based on a £9 hourly rate and bonus compensation dependent on the result of the trust game (up to 55% of the base compensation).

### Procedure

The participants were first instructed about the rules of the game and asked to report how well they understood them. Then, they picked the other player to interact with during the game from a pool of eight players represented in randomized order with one of the first eight letters of the alphabet (Player A through Player H). After choosing the other player, participants read their short description, which contained experimental manipulations, and answered manipulation check questions (see details in the next section). Finally, the participants played one round of the trust game, which constituted a behavioral measure of trust towards the trustee.

### Experimental manipulations

Experimental manipulations of the trustee type (type of trustee: AI agent vs. human), trustee warmth (low vs. high), and trustee competence (low vs. high) were embedded in the description of the other player. In the human trustee conditions, the other player was introduced as “a human,” while in the AI agent trustee conditions, they were introduced as “an AI agent.” In the high warmth conditions, the other player was referred to as “partner” who “wants what’s best for you,” while in the low warmth conditions, the other player was referred to as “opponent” who “wants what’s best for them.” In the high competence conditions, the other player’s skills in the game were rated at “10/10”, while in the low competence conditions, they were rated at “1/10”. We used the following description of the other player: “Your partner [opponent] is a human [AI agent]. They want what’s best for you [them]. Your partner’s [opponent’s] skills in this game are 1/10 [10/10]”. Participants were randomly assigned to one of the experimental conditions.

To verify the effectiveness of experimental manipulation of trustee warmth and competence, we used three manipulation check items: “Based on received information, the other player is: (a) friendly; (b) trustworthy; (c) competent”, on a Likert-type scale ranging from 1—not at all to 7—completely. Items (a) and (b) were used to measure the effectiveness of the warmth manipulation, while item (c) was used to measure the effectiveness of the competence manipulation.

### Trust game

We used a typical trust game setup^39^ and standardized game instructions^41^ adapted to this study: “This task is about an exchange between yourself and another player whom you will pick. You do not know this other player and you will not knowingly meet them. You have been randomly assigned the role of the “sender.” The other person is in the role of the “receiver.” You and the receiver are both endowed with 10 tokens. You first decide how much of your 10 tokens endowment to transfer to the receiver. You can choose any amount between 0 tokens and 10 tokens. The amount you transfer is tripled (by the experimenter) before being received by the receiver. The amount you keep is not tripled and simply remains in your possession. The receiver then decides how much of the tripled transfer to return to you. The receiver can choose any amount between 0 tokens and the sum of their endowment and the tripled amount you sent. The amount the receiver returns is not tripled. Your final payment is the sum of the amount of your 10 tokens endowment you keep and the amount the receiver returns. Each token you have at the end of the game is worth £0.01 bonus payment. The receiver’s final payment is what they have left after they make the transfer to you (or not).” The amount sent to the other player was our second dependent variable.

We ensured that participants understood the rules of the trust game by asking them: “How well do you understand the rules of the game?” on a Likert-type scale from 1—not at all to 7—completely.

## Results

### Trust game rules

The participants understood the rules of the trust game very well (M = 6.54, SD = 0.83, on a scale from 1 to 7).

### Manipulation checks

Two manipulation check items measuring the effectiveness of the trustee warmth manipulation (i.e., perceived friendliness and trustworthiness of the trustee) were highly correlated, r (399) = 0.79, *p* < .001 and we averaged them to obtain a single manipulation check measure of the trustee warmth. Trustee warmth and trustee competence manipulations both proved effective. In the high-warmth conditions, trustees were perceived as warmer (M = 4.91, SD = 1.52) than in the low-warmth conditions (M = 3.01, SD = 1.75), t(325.61) = 11.30, *p* < .001, and in the high-competence conditions trustees were perceived as more competent (M = 5.76, SD = 1.49) than in the low-competence conditions (M = 3.14, SD = 1.96), t(336.04) = 14.87, *p* < .001.

### Hypotheses testing

**Fig. 1:**
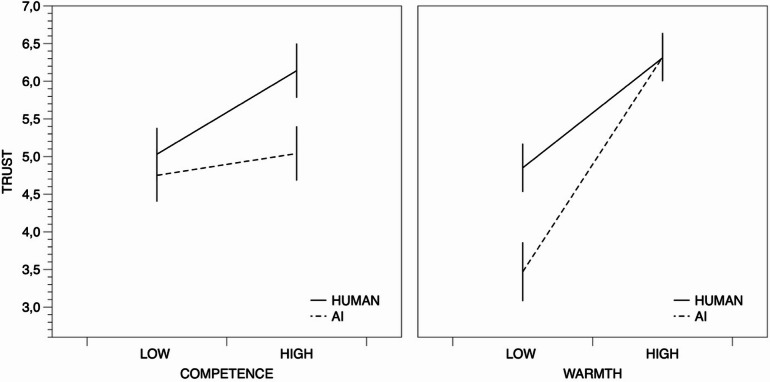
Trust towards human and AI trustees, depending on trustee competence (low vs. high) and trustee warmth (low vs. high). Error bars represent standard errors of the means.

To verify the effect of trustee type, trustee warmth, and trustee competence on participants’ behavior in trust game, we conducted a univariate analysis of variance with the type of trustee (type of trustee: AI agent vs. human), trustee warmth (low vs. high) and trustee competence (low vs. high) as fixed factors and allocations made in trust game as the dependent variable (see Fig. 1).

All three main effects were significant, type of trustee F(1,392) = 4.26, *p* = .04, partial eta = 0.01, trustee warmth F(1,392) = 41.97, *p* < .001, partial eta = 0.10, and trustee competence F(1,392) = 4.39, *p* = .04, partial eta = 0.01. Human trustees (M = 5.58, SE = 0.22) were trusted more than AI agent trustees (M = 4.89, SE = 0.25). High-warmth trustees (M = 6.31, SE = 0.22) were trusted more than low-warmth trustees (M = 4.16, SE = 0.25). Finally, high-competence trustees (M = 5.56, SE = 0.23) were trusted more than low-competence trustees (M = 4.89, SE = 0.24).

The interaction between the type of trustee and trustee warmth was significant, F(1,392) = 4.44, *p* = .04, partial eta = 0.01. Simple effects tests showed that in the high-warmth conditions, trust towards human trustees (M = 6.31, SE = 0.29) and towards AI trustees (M = 6.32, SE = 0.32) was not significantly different, F(1,392) = 0.001, *p* = .98, but in the low-warmth conditions trust towards human trustees (M = 4.85, SE = 0.32) was higher than trust towards AI agent trustees (M = 3.47, SE = 0.39), F(1,392) = 7.48, *p* = .01, partial eta = 0.02. Both human and AI agent trustees were trusted more in the high-warmth than in the low-warmth conditions, F(1,392) = 11.18, *p* < .001, partial eta = 0.03 and F(1,392) = 31.17, *p* < .001, partial eta = 0.08, respectively.

The interactions between type of trustee and trustee competence, trustee warmth and trustee competence, as well as the three-way interaction between type of trustee, trustee warmth and trustee competence were not significant, F(1,392) = 1.55, *p* = .21, F(1,392) = 0.07, *p* = .80, F(1,392) = 0.24, *p* = .63, respectively.

## Discussion

The present work investigated the effects of trustee warmth and competence on trust expressed towards them in a trust game, comparing AI agent trustees with human trustees. It seems to make a worthwhile contribution to the understanding of how trustee’s characteristics affect trust towards AI agents in human-AI interactions.

Our results showed that warmth and competence affect trust towards both AI agent and human trustees. For both types of trustees, trust was higher in the case of high-competence (vs. low-competence) and high-warmth (vs. low-warmth) trustees. However, when we compared the levels of trust between human-human and human-AI interactions, we found two important differences. Firstly, AI trustees were, on average, trusted less than humans. Secondly, while competence had a similar effect on trust towards both types of trustees, the effect of warmth on trust was much stronger in the case of AI trustees than in the case of human trustees. Low warmth AI trustees were the least trusted among all the trustees in the study.

We propose to interpret our present findings in the context of worries in response to the development of AI technologies. One of the fears associated with AI is that these technologies could eventually turn against humans and threaten humanity’s existence^6,11,13^. This fear has been fueled by technological developments, concerns voiced by experts, as well as science fiction narratives. Put in terms of social cognition theory, people are especially afraid of low-warmth AI, i.e., AI agents with their own goals, which do not necessarily align with those of the perceiver. Therefore, a technology oriented at its own objectives elicits strong negative affective attitudes and is hence trusted less. Recent research^30^ has reinforced the critical role of value alignment in human-AI interactions. In this study, participants engaged in a social dilemma game, Coins, with an artificial agent trained to exhibit either individualistic (low value alignment) or prosocial (high value alignment) behavior. Agents that acted prosocially were perceived as warmer, described using more positive language, and preferred as interaction partners compared to their individualistic counterparts. Interestingly, participants favored agents perceived as incompetent over those perceived as competent, a finding that may reflect a preference for prosociality—particularly if selfishness was implicitly associated with competence. In future studies, we plan to investigate whether the relationship between value alignment and perceived trustworthiness/trust can be explained by this existential fear related to AI technologies.

The implications of the present work are geared towards practical recommendations for designing trustworthy AI technologies. Our results show that, first of all, the degree to which the AI technology is perceived as oriented towards the user’s (vs. its own) goals drives the perception of its warmth and, in consequence, trust towards it. Secondly, it seems that the perceptions of low warmth of AI technologies can shatter the trust towards them, independently of how well these technologies perform. Therefore, people could be apprehensive of technologies that may be perceived as oriented at pursuing their own agenda, no matter how well these technologies could actually assist the people in achieving their goals. Based on the results of the present research, we suggest that increasing the perceived warmth of AI technologies, i.e., the perceived correspondence of interests between the technology and the user, should lead to greater trust towards them.

Several limitations should be considered when interpreting our findings. The main limitation of the presented research is its generalizability due to the specificity of the domain (trust game) and AI agent type (the other game player). Trust game is an ingenious experimental paradigm that has delivered important insights and remains a benchmark in trust research. It has been shown to systematically predict self-reported trust, even when controlling for other social preferences as well as for a broad range of individual characteristics^40^. However, it is too stylized to capture the complete picture of the nuances of trust and isn’t always predictive of real-world behavior, especially in the context of more complex human-AI systems involving dynamic or reciprocal interactions, such as collaborative work or decision-making in high-stakes environments^42,43^. More research in contexts of varying complexity and using different measures would contribute to making the results more generalizable and more ecologically valid.

Another limitation is participant sampling. The study’s sample consisted of individuals volunteering for research tasks through the online platform Prolific, which may result in a sample primarily consisting of individuals who possess greater IT proficiency than the average population. While being conducted on a representative sample undoubtedly constitutes a strength of the presented research, it is linked to another limitation, namely cultural homogeneity. The US is a typically individualistic culture that refers mostly to competence, in contrast to collectivistic cultures that refer mostly to warmth^44,45^. It is thus unclear whether obtained results could be generalized to collectivistic cultures. More cross-cultural research would be needed to corroborate obtained results.

## Data Availability

The dataset generated for this research can be found in the Figshare repository under the terms of the Creative Commons Public Domain license with DOI 10.6084/m9.figshare.26139574.
